# Investigation of the biological functions of heparan sulfate using a chemoenzymatic synthetic approach

**DOI:** 10.1039/d0cb00199f

**Published:** 2021-02-22

**Authors:** Zhangjie Wang, Katelyn Arnold, Vijay Manohar Dhurandhare, Yongmei Xu, Jian Liu

**Affiliations:** Division of Medicinal Chemistry and Medicinal Chemistry, Eshelman School of Pharmacy, University of North Carolina Chapel Hill North Carolina USA jian_liu@unc.edu

## Abstract

Heparan sulfate (HS) is a highly sulfated polysaccharide playing essential physiological and pathophysiological roles in the animal kingdom. Heparin, a highly sulfated form of HS, is a widely used anticoagulant drug. Isolated from biological sources, both heparin and HS are polysaccharide mixtures with different sugar chain lengths and sulfation patterns. Structural heterogeneity of HS complicates the investigation of HS-related biological activities. The availability of structurally defined HS oligosaccharides is critical in understanding the contribution of saccharide structures to the functions. The chemoenzymatic synthetic approach is emerging as a cost-effective method to synthesize HS oligosaccharides. Structurally defined oligosaccharides are now widely available for biologists. This review summarizes our efforts in using this new synthetic method to develop new anticoagulant therapeutics and discover the role of HS to protect liver damage under pathological conditions. The synthetic method also allows us to prepare reference saccharide standards to improve structural analysis of HS.

## Abstract 2

Heparan sulfate (HS) is a highly sulfated polysaccharide playing essential physiological and pathophysiological roles in the animal kingdom.

## Graphical abstract

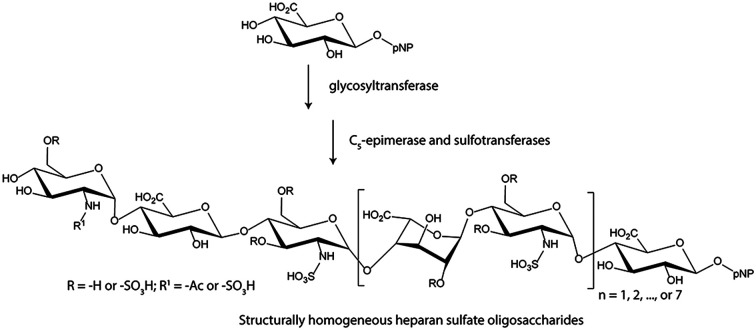

Heparan sulfate (HS) is a sulfated polysaccharide present on the cell surface and in the extracellular matrix. HS exists in the form of HS proteoglycan (HSPG) that contains a core protein and HS polysaccharide side chains. The biological functions of HS proteoglycans are largely dominated by HS side chains. HS plays important roles in the innate immune system including aiding in leukocyte extravasation,^1^ interacting with chemokines, and mediating reactive molecules released by neutrophils^2^ (Fig. 1). HS facilitates the interactions between fibroblast growth factor (FGF) and FGF receptor to regulate cell proliferation.^3^ It also serves as a receptor for herpes simplex virus entry, allowing the virus to bind to the host cells to establish the infection.^4^ Antithrombin (AT), a protease inhibitor regulating the blood coagulation cascade, binds to HS. The complex of AT and HS inhibits the proteolytic activities of thrombin (factor IIa) and factor Xa to prevent blood from clotting.^5^ Heparin, an analog of HS, has been widely used in hospitals to treat blood clotting disorders for nearly a century. There are considerable interests among researchers to expand the potential therapeutic in curbing inflammatory responses and antiviral properties. HS consists of the disaccharide-repeating units of glucuronic acid (GlcA) or iduronic acid (IdoA) linked to a glucosamine. Both IdoA and glucosamine residues frequently carry sulfo groups. Locations of the sulfo groups and positions of GlcA and IdoA residue determine the functions of HS. Recent advancement in the synthesis of structurally homogeneous HS oligosaccharides makes the compounds available. The availability of HS oligosaccharides offers the opportunity to discover new HS-based therapeutics. This article intends to review the current progress based on the synthesis of HS oligosaccharides using the chemoenzymatic synthesis method.

**Fig. 1:**
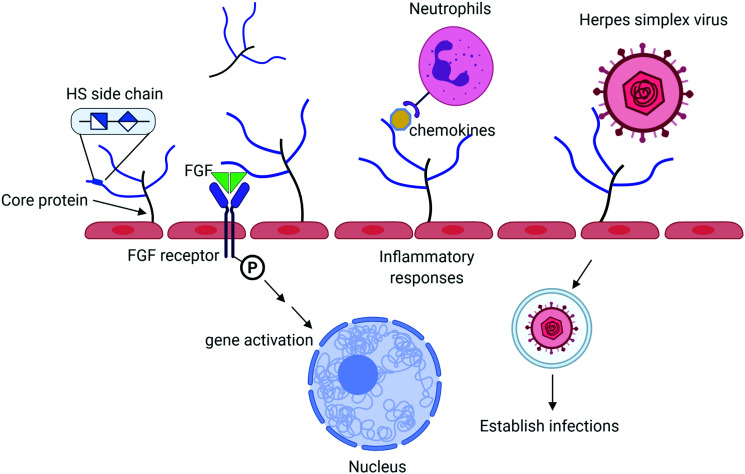
Summary of biological functions of HS. HS proteoglycan (HSPG) consists of a core protein and HS polysaccharide side chains. HS chains interact with antithrombin to regulate blood coagulation process and to fibroblast growth factor (FGF) and FGF receptor to impact cell proliferation. HS also bind to chemokines to recruit leukocytes in response to inflammation. Herpes simplex virus (and other viruses) use HS on the cell surface to establish the infections. HSPG is one of the major components of endothelial glycocalyx, implementing in many other functions as discussed in the text.

## HSPG and the endothelial glycocalyx

### Endothelial glycocalyx

A single layer of endothelial cells lines every blood vessel in the vascular system.^1^ The glycocalyx covers the luminal side of endothelial cells, separating the endothelium from direct contact with blood flow.^2^ The glycocalyx is rich in carbohydrates and is comprised of proteoglycans, glycoproteins, glycosaminoglycans, and plasma proteins. The gel-like layer of the glycocalyx is essential for normal physiology in several aspects: it regulates the permeability of the vasculature, serves as a mechanotransducer, and functions as an anti-inflammatory and anti-coagulant coat to protect the underlying endothelium.

The glycocalyx is described as a macromolecular sieve that regulates molecular and fluid trans vascular flux.^6,7^ It is well known that in pathological conditions, like sepsis, atherosclerosis, and kidney disease, the glycocalyx is damaged resulting in vascular hyperpermeability and increased apoptosis.^8–11^ Heparanase, a HS degrading enzyme, is frequently associated with glycocalyx damage. Loss of any glycocalyx component, especially the dominating presence of HS, leads to deterioration of glycocalyx integrity and function. In a bacterial lipopolysaccharide (LPS)-induced sepsis mouse model, pulmonary endothelial heparanase was activated in a TNF-α dependent manner.^12^ Consequently, the increase in heparanase activity leads to the development of acute lung injury during endotoxemia.^12^ In that study, the authors also demonstrate the enhanced diffuse alveolar damage by heparanase from biopsied human lung tissue. The findings raise a possible approach to preserve the integrity of endothelial glycocalyx through inhibiting the activity of heparanase to prevent the degradation of HS.

### HS metabolism

HS preparations isolated from cells or tissues are complex mixtures of polysaccharides and are covalently attached to the core protein in the form of HSPGs. The syndecan family, including Syndecans 1–4, is the most common HSPGs on the cell surface, where the core protein from syndecan is anchored on the membrane. The core protein from each syndecan isoform has a unique amino acid sequence, while the structural difference in HS side chains among syndecans is not well established. The core proteins are subjected to proteolytic cleavage under pathophysiological conditions, a process known as syndecan shedding.^11,13^ HS metabolism is carried out in lysosomes involving in a number of sulfatases and glycosidases. The concerted actions of the sulfatases and glycosidases reduce long HS sugar chains into individual monosaccharides. The deficiency of enzymes for degrading HS in lysosomes leads to the mucopolysaccharides clinic features due to the accumulation of HS polysaccharides in multiple organs.^14^ The elevated HS as a result of syndecan shedding or abnormal catabolism of HS in lysosomes circulate into fluids such as blood, cerebrospinal fluid or urine.^15^ Determination of structure and quantity of HS in biological fluids or tissues may enable HS a promising biomarker for prognosis assessment.

### HS interactions with chemokines

Chemokines are central to the innate immune system and participate in the recruitment of leukocytes to inflammation tissues. Chemokines are a type of chemotactic cytokines and are basic in nature, which leads to tight binding to negatively charged glycosaminoglycans, especially HS.^16^ HS and chemokine interactions protect chemokines from proteolysis,^17^ present chemokines to their receptors,^18^ establish chemotactic gradients,^18,19^ and participate in oligomerization of some chemokines.^20,21^ With technological advances in synthesizing and detecting specific HS oligosaccharides, studies have been able to discern specific HS/chemokine interactions, thus supporting the notion that these interactions are not solely electrostatic.^22^

In a recent study by van Gemst, *et al.*, a panel of anti-HS single chain variable fragment antibodies specific for distinct HS modifications were used to probe the HS structural specificity for binding to recombinant murine chemokines CXCL1, CXCL2, and CCL2 to mouse glomerular endothelial cells.^22^ They found that an antibody that binds to HS domains displaying full sulfation significantly inhibited chemokine binding to the cell surface for all chemokines studied. Concurrently, they demonstrated that the enoxaparin, a low molecular weight heparin drug approved by FDA, displaced CXCL2 and CXCL1 from binding to the cell surface. The results suggest that both CXCL2 and CXCL1 bind to the oligosaccharides from the enoxaparin that contains a wide range of sulfation patterns, including 3-*O*-sulfation.

HS binding to chemokines has been assessed using a HS microarray comprised of 47 tetrasaccharides synthesized through a chemical approach.^23^ This study reveals structural insights into the binding of CCL2, CCL7, CCL5, CCL13, CXCL8, and CXCL10 to HS tetrasaccharides. While endogenous HS is much longer than a tetrasaccharide, this microarray provides a useful resource to begin to investigate potential important structural determinants. For example, this study demonstrated that a higher level of sulfation did not correlate to increased binding for CXCL8, CXCL10, and CXCL13. Furthermore, compounds with 3*-O*-sulfation showed interesting binding trends. CCL2, CCL5, CCL7, and CCL13 had the highest response to GlcA-GlcNS3S-IdoA2S-GlcNS6S. However, exchanging the 3-*O*-sulfation modification for 6-*O*-sulfation on the same residue (GlcA-GlcNS6S-IdoA2S-GlcNS6S), greatly diminished the signal. In fact, this 6-*O*-sulfated compound, had a similar or decrease signal for all chemokines, except CCL2, compared to compounds with only one sulfation present. These results suggest that the placement of sulfation modifications in the oligosaccharide rather than the degree of overall charge is important for chemokine binding. Further understanding of the preferred HS substrates may lead to therapeutics with the ability to selectively target certain chemokines.

## Chemoenzymatic synthesis of HS

### Synthetic scheme

One challenge in the HS research is to make the structurally defined HS oligosaccharides. Structurally homogenous HS oligosaccharides are very difficult to synthesize using chemical methods as the synthetic routes are long. Enzymatic and chemoenzymatic syntheses of HS are becoming promising alternatives to access structurally defined oligosaccharides, especially for longer than hexasaccharides. Long HS chains are required for investigating the biological functions.

**Fig. 2:**
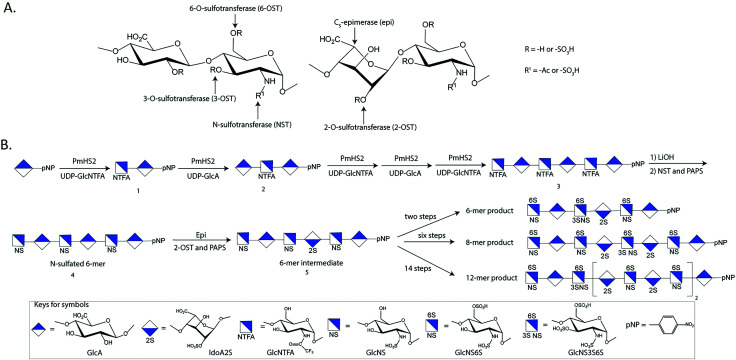
Chemoenzymatic scheme for the synthesis of ULMW and LMW heparins. Panel A shows the structures of disaccharide repeating units of HS. The biosynthesis of HS is completed through four sulfotransferases and one C5-epimerase. The action site for each sulfotransferase and the C_5_-epimerase is indicated. Panel B shows the synthetic steps for the synthesis of anticoagulant 6-mer, 8-mer and 12-mer products. These oligosaccharides have unique properties as a synthetic heparin candidate. The synthesis started from monosaccharide 1-*O*-(*p*-nitrophenyl) glucuronide (GlcA-pNP), which is commercially available. The recovery yield at each synthetic step is indicated below the reaction arrow. Abbreviations: NST, *N*-sulfotransferase; PAPS, 3′-phosphoadenosine 5′-phosphosulfate; PmHS2, heparosan synthase 2 from *Pasteurella multocida*.

The chemoenzymatic approach involves the use of glycosyltransferases to build the saccharide backbone, one C_5_-epimerase (epi) to convert a GlcA residue to an IdoA residue, and four different sulfotransferases to install the sulfo groups to specific –OH positions of the glucosamine or GlcA and IdoA residues. One key reagent used in the steps to build saccharide backbone is UDP-GlcNTFA (uridine diphosphate *N*-trifluoroacetylated glucosamine), an unnatural sugar nucleotide. The GlcNTFA is incorporated into the sugar backbone from UDP-GlcNTFA to form **1** by PmHS2, heparosan synthase 2 from *Pasteurella multocida* (Fig. 2B). The GlcNTFA residue is readily converted to a GlcNS residue after detrifluoroacetylation followed by *N*-sulfation using *N*-sulfotransferase (NST) to form **4**. This approach controls the position for GlcNS residue. The oligosaccharide is subjected to the enzymatic modifications by 2-*O*-sulfotransferase (2-OST), C_5_-epimerase (epi), 6-*O*-sulfotransferase (6-OST) and 3-*O*-sulfotransferase (3-OST) to form the HS oligosaccharides with elaborated sulfation patterns. The method has been demonstrated in synthesizing HS oligosaccharides^24^ with biological and pharmaceutical applications. Rearranging the sequence of the enzymatic modification steps is essential for synthesizing some oligosaccharides due to the substrate specificities of HS biosynthetic enzymes, *i.e.* the oligosaccharides containing multiple IdoA2S residues.^25^ Extensive reviews for the chemoenzymatic method for the synthesis of HS have been published elsewhere.^5,26^ The chemoenzymatic synthesis reportedly produces a library of HS oligosaccharides consisting of 90 individual oligosaccharides for biological research,^24^ and the actual available number of oligosaccharides is growing.

The chemoenzymatic synthetic method is used to synthesize structurally heterogeneous polysaccharides.^27,28^ The synthesis follows a very similar route, but the elongation steps are omitted. Although the polysaccharides are still mixture in size, the synthesis process is used to control the sulfation patterns. The methods for the chemoenzymatic synthesis of polysaccharides and the utilities are reviewed elsewhere.^26^

### Engineering the substrate specificity of enzymes to expand chemoenzymatic synthesis

One limitation for the chemoenzymatic method is the range of oligosaccharides generating from strict substrate specificity of sulfotransferases. High substrate regioselectivity from sulfotransferases reduces by-products formation thereby increases the synthetic efficiency. But this property imposes restrictions on the number of compounds with diverse HS oligosaccharides. A potential solution is to engineer HS biosynthetic enzymes to cover the synthesis of additional saccharide sequences. Tinkering the amino acid residues of the substrate binding site allows the mutated enzyme to synthesize the saccharide sequences beyond the capability of the wild type enzyme. As an example, we demonstrated that a 6-OST-3 mutant, 6-OST-3 R112E/R206E/R329E, adds additional selectivity to introduce the 6-*O*-sulfation.^29^ Three positively charged amino acid residues, including R112, R206 and R329, are used by the enzyme to balance the negative charge from substrates or products with multiple 6-*O*-sulfo groups. Substituting these positively charge amino acid residues with negatively charged residues removes the enzyme's ability to transfer multiple 6-*O*-sulfo groups to an oligosaccharide substrate. The 6-OST-3 mutant, unlike its wild type protein, only sulfates the glucosamine residue on the non-reducing end. The 6-OST-3 mutant expands the ability to synthesize partially 6-*O*-sulfated oligosaccharides.^29^ Numerous crystal structures of other HS sulfotransferases have been reported, revealing how the enzymes interact with substrates.^30–33^ The structural information will guide engineering efforts to create the sulfotransferases with desired substrate specificities.

### Example 1: Preparation of synthetic heparin candidates to replace animal sourced heparin

Chemoenzymatic method has been employed to prepare synthetic heparin, HS-based therapeutics. Heparin is a highly sulfated form of HS. Discovered in late 1910's, heparin plays significant roles in the development of surgery and kidney dialysis. Pharmaceutical heparin is a mixture of polysaccharides with different sizes and sulfation patterns. Heparin is a product isolated from pig intestine through a long and poorly regulated supply chain.^34,35^ Batches of contaminated heparin entered to the marketplaces in 2007–2008, resulting in 84 deaths in US.^36^ There has been a strong demand for a cost-effective method to prepare synthetic heparin to substitute animal sourced heparin to improve the safety of the drug.^37^ The chemoenzymatic strategy is becoming a promising method to prepare cost-effective synthetic heparin to substitute animal-sourced counterparts.^5^

Three forms of heparin are approved by the US FDA, including unfractionated heparin (UFH), low-molecular weight heparin (LMWH) and fondaparinux. UFH is isolated from porcine intestine, and LMWH is a depolymerized unfractionated heparin. Thus, both UFH and LMWH are animal-sourced materials. Fondaparinux is a fully synthesized single molecular entity of pentasaccharide. But its pharmacological properties are distinct from UFH and LMWH, fondaparinux is thereby not a substitute for heparin. The chemoenzymatic method can synthesize short oligosaccharides, like fondaparinux, as well as long oligosaccharides matching the size and pharmacological properties of LMWH. The approach was first employed in the synthesis of two heptasaccharide constructs, fondaparinux-like oligosaccharides.^38^ Chemoenzymatic synthesis has significantly shortened the synthesis, *i.e.* from ∼50 steps to ∼10 steps and improved final yields from ∼0.1% to ∼40%.^35,38^ The heptasaccharide has now been replaced by a 6-mer product for further development (Fig. 2B). This synthetic approach was next used to prepare a dodecasaccharides (12-mer), a LMWH analog (Fig. 2B).^25^ The anticoagulant activity of 12-mer showed complete neutralization with protamine, an FDA approved drug used as an antidote for unfractionated heparin.^25^ An anticoagulant 8-mer product was also synthesized. The property of the 8-mer product is that the compound has fast clearance from the body as demonstrated in the rat model, offering a unique clinical advantage for in-hospital patients that are vulnerable to bleeding (Fig. 2B).^39^

The scalability of chemoenzymatic synthesis has been a concern. We have recently demonstrated the synthesis of 1.4 g of the fully sulfated 12-mer using the chemoenzymatic method.^40^ Commercial scale-up synthesis is underway with a goal to advance synthetic heparin to the market.

### Example 2. Investigation of the sulfation patterns to the conformation of IdoA and IdoA2S residues

The conformation of HS is a factor to determine the interaction with specific protein targets.^27^ For example, the conformation of l-iduronic acid (IdoA) residue (^1^*C*_4_-chair and ^2^*S*_0_-skew boat) plays a critical role in determining the interaction between antithrombin and HS.^41^ Homogeneous HS oligosaccharides allow us to investigate the impact of 2-*O*-, 3-*O*- and 6-*O*-sulfation on the conformation dynamics change of IdoA. The chemoenzymatic synthesized HS oligosaccharides contributed to investigate how the saccharide sequence to the conformation of a series of HS hexasaccharides.^42^ Interestingly, the data from NMR analysis indicated the IdoA2S residue adapts its conformation from ^1^*C*_4_-chair to ^2^*S*_0_-skew boat when the level of sulfation from the flanking saccharide residues increases; whereas the IdoA residue adapts its conformation from ^2^*S*_0_-skew boat to ^1^*C*_4_-chair when the level of sulfation from the flanking saccharide residues increases. It should be noted that the energy barrier between conformations of ^1^*C*_4_-chair and ^2^*S*_0_-skew boat is small. Both conformations frequently co-exist in solution if oligosaccharides do not bind with proteins.

### Example 3: Synthesis of stable isotopically labeled HS standards for quantitative disaccharide analysis

**Fig. 3:**
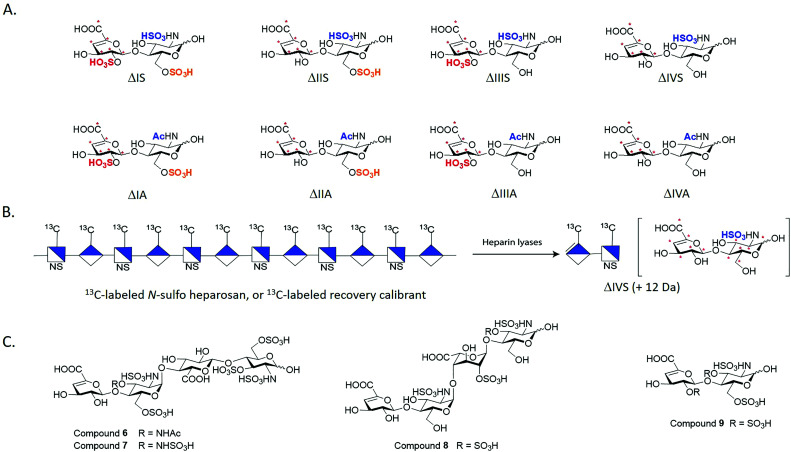
Structures of synthetic disaccharides, polysaccharide and tetrasaccharides for structural analysis. Panel A shows eight ^13^C-labeled HS disaccharides. These disaccharides are used for the disaccharide analysis of HS. “*” indicates the ^13^C-labeling site. The disaccharides are prepared from oligosaccharides carrying site-specifically labeled ^13^C-labeled GlcA residues. Panel B shows the schematic structure of ^13^C-labeled *N*-sulfo heparosan that is used as a recovery calibrant. The polysaccharide is susceptible to the degradation of heparin lyases to yield disaccharide ΔIVS, but its molecular mass is 12 Da higher than the native counterpart. Panel C shows the structures of three 3-*O*-sulfated tetrasaccharides and one 3-*O*-sulfated disaccharide.

We employed the chemoenzymatic method to prepare eight ^13^C-labeled disaccharides that are found in HS to add the ability to quantify from organs, tissues and cells. One common approach for structural analysis is to degrade HS polysaccharides to disaccharides using heparin lyases I, II and III, leading to disaccharides carrying an Δ_4,5_-unsaturated uronic acid at the non-reducing end (Fig. 3A).^43,44^ These disaccharides are readily resolved by HPLC or capillary electrophoresis for the compositional analysis. A variety of separation techniques coupled with electrospray ionization (ESI)-MS or MS/MS as detector has been widely used for HS disaccharide characterization. Several separation approaches including hydrophilic interaction liquid chromatography (HILIC), reverse-phase ion pairing (RPIP), porous graphitic carbon (PGC) and size exclusion chromatography (SEC) are compatible with ESI-MS.^45^ In order to improve the detection sensitivity, the label reagents have been introduced into the reducing end of disaccharides through reductive amination. The chemical label also increases the ionization efficiency and enhance resolution among different disaccharides on a reverse phase C18 column.^46,47^

Implementing isotopically labeled standards to LC/MS based disaccharide analysis, or isotope dilution mass spectrometry, should improve the accuracy and precision of quantification of HS. Isotopically labeled authentic internal standards are particularly essential for quantitative analysis because that they are chemically equivalent to the native analyte and co-eluted on the HPLC column. The use of internal standards circumvents experimental bias introduced by extraction efficiency, HPLC injection, ionization efficiency and matrix component effects.^48,49^ One example is to introduce deuterated aniline to disaccharides by reductive amination. The isotopically labeled aniline/disaccharide conjugates were then used as internal standards.^50,51^ The accurate quantitation is based on the assumption that the coupling reaction to prepare aniline/disaccharide is complete during the analysis. An alternative strategy to insert stable isotopically saccharides by chemoenzymatic synthesis has been introduced.^44,52^ The isotopically labeled heparosan polysaccharides carried with different sulfation patterns were produced by the expression of *E. coli K5* on ^13^C glucose and ^15^N ammonium chloride, followed by several sulfotransferases modification to prepare heparin-like polysaccharides. The resultant isotopically labeled HS disaccharide internal standards were obtained by heparin lyases depolymerization.^52^ However, two disaccharides, ΔUA2S-GlcNAc (ΔIIIA) and ΔUA2S-GlcNAc6S (ΔIA), were absent from this study. Both ΔIIIA and ΔIA are more difficult to isolate from lyases digested heparin-like polysaccharides because they are low abundant.

We took the advantage of chemoenzymatic synthesis approach to synthesize a series of oligosaccharides embed with ^13^C-labeled GlcA/IdoA2S residues at desired sites. These oligosaccharides were then digested with heparin lyases to prepare authentic eight ^13^C-labeled disaccharides in large quantity (Fig. 3A). Each disaccharide standard has an increase of 6 Da in molecular mass compared with native disaccharides.^44^ We also prepared a ^13^C-labeled *N*-sulfo heparosan, known as recovery calibrant, to be added in the analysis (Fig. 3B). The ^13^C-labeled recovery calibrant is added as early as extraction process start to control the recovery yield of the HS sample from the extraction process.^44^ The recovery calibrant is also susceptible to the digestion of heparin lyases to yield a disaccharide △UA-GlcNS (ΔIVS). The ΔIVS disaccharide from recovery calibrant carries 12 Da higher molecular mass than native △IVS and 6 Da higher than ^13^C-labeled internal standard ^13^C-labeled △IVS (Fig. 3B). The method enables finishing disaccharide quantification and recovery yield calculation in one-pot process.^44^ The use of a combination ^13^C-labeled disaccharides and ^13^C-labeled recovery calibrant has achieved absolute quantitative analysis of HS from mouse tissues.^44^ Notably, chemoenzymatic synthesis of individual ^13^C-labeled disaccharides can be accomplished in tens of milligrams, including low abundant disaccharides such as ΔIIIA and ΔIA.

As the rarest sulfation in HS, 3-*O*-sulfation is linked to biological functions including anticoagulant activity, binding of FGF receptors and viral glycoprotein D of herpes simplex virus to establish the infection. Heparin consists of an antithrombin-binding pentasaccharide sequence and contains a 3-*O*-sulfated glucosamine residue critical for the anticoagulant activity. Using the oligosaccharides and heparin lyases, we also prepared 3-*O*-sulfated tetrasaccharide standards (**6–8**, Fig. 3C) and a 3-*O*-sulfated disaccharide (**9**, Fig. 3C) that have Δ_4,5_-unsaturated uronic acid reside at the non-reducing end.^53^ These oligosaccharides resemble, in part, those released from heparin lyases degraded pharmaceutical heparin or 3-*O*-sulfated HS isolated from biological tissues.^54^ Unlike other oligosaccharides, the 3-*O*-sulfated glucosamine residue at the non-reducing end is labile under basic conditions. The instability of the 3-*O*-sulfated tetrasaccharides (6–8) and the 3-*O*-sulfated disaccharide (9) potentially adds complexity for accurately measuring the amount of 3-*O*-sulfation in HS from biological sources.

### Example 4: Design of anti-inflammatory HS to protect liver against pathological damages

Sterile inflammation is a natural response by the immune system to cellular damage in the absence of bacteria or viruses. However, dysregulated and exuberated inflammatory response may cause excessive damage to the neighboring tissues. Sterile inflammation is a major contributor to many disease states. The immune system contains pattern recognition receptors (PRRs) which sense pathogen-associated molecular patterns (PAMPs) in microorganisms. PRRs can also recognize damage associated molecular patterns (DAMPs) which are endogenous molecules released during sterile inflammation.^55^ DAMPs include heat shock proteins, S100 proteins, histones and high mobility group box-1 (HMGB1).^55^ DAMPs are characterized as molecules that are necessary for normal physiology yet act as a danger signal when their typical localization is disrupted. For example, HMGB1 and histones reside in the nucleus attached to DNA and are released when the cell undergoes necrosis. These intracellular components found outside of the cell signal to the immune system that damage is occurring. Interestingly, HS is considered DAMPs in some contexts when released from degrading extracellular matrix (ECM) during tissue injury.^55^ However, a direct implication of endogenous HS causing sterile inflammation *in vivo* has not been demonstrated.

Many DAMPs that bind to toll-like receptors (TLRs) are PRRs, and elicit an inflammation response. Some DAMPs, including HMGB1, also bind to the receptor for advanced glycation end products (RAGE) which is important in sterile inflammation but not clearly characterized in pathogenic inflammation. Notably, many DAMPs are also HS binding proteins so understanding the role of DAMPs in sterile inflammation and the relationship with HS will reveal HS's function in sterile inflammation and may identify targets for HS-based therapeutics in cases of sterile inflammation.

### Synthetic HS oligosaccharides protect against acute liver injury caused by acetaminophen overdose

**Fig. 4:**
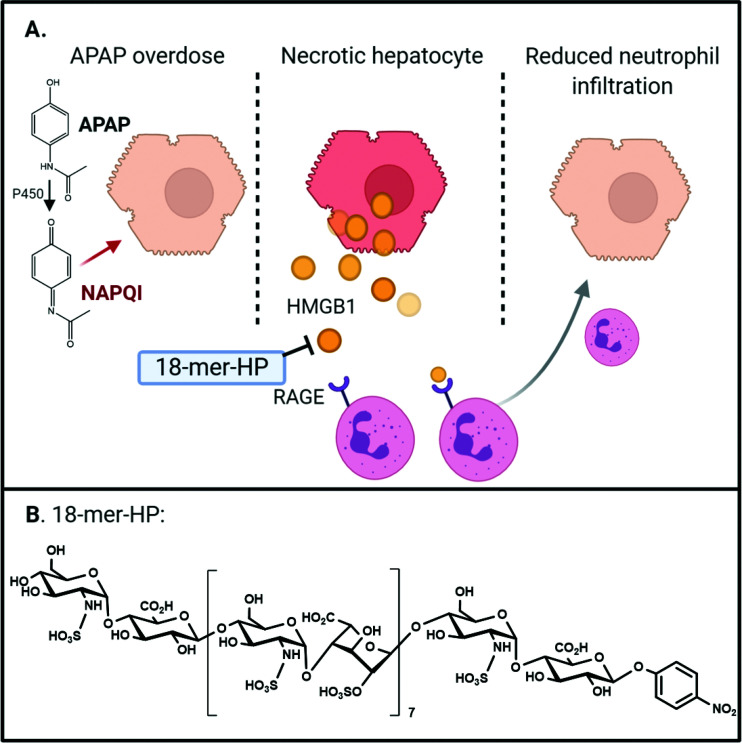
Anti-inflammatory HS 18-mer HP protects against liver damage caused by acetaminophen overdose. Panel A shows the mechanism of action of 18-mer HP. Left panel shows that acetaminophen (APAP) is converted to a reactive metabolite, *N*-acetyl-*p*-benzoquinone imine (NAPQI) by P450 enzyme in the liver. Middle panel shows that NAPQI causes hepatocyte necrosis leading to DAMP release. DAMPs, *i.e.* HMGB1, recruit neutrophils to the injury site. This dysregulated response causes continued hepatocyte necrosis and inflammation. 18-mer-HP binds to HMGB1 and neutralizes its pro-inflammatory activity. The right panel shows that neutralization of HMGB1 by 18-mer-HP attenuates neutrophil infiltration, reducing the injury damage caused by dysregulated inflammatory response. Panel B shows the chemical structure of 18-mer-HP.

Acetaminophen (APAP), also known as paracetamol, is a widely used analgesic and is the active pharmaceutical ingredient of Tylenol®. Although APAP is generally safe under the recommended dose, overdose of APAP leads acute liver failure (ALF),^56^ which is a leading cause for drug-induced liver injury in US and Europe. The misuse of Vicodin® or Percocet®, co-formulations of opioids and APAP, can also cause ALF. In the US, nearly 50% of drug-induced liver injury has been attributed to APAP toxicity,^57^ which accounts for ∼80 000 emergency room visits annually.^58^ The mechanism for APAP toxicity begins with its metabolic conversion to the reactive chemical species, *N*-acetyl-*p*-benzoquinone imine (NAPQI), which causes hepatocyte necrosis.^59^ In an elegant study by Huebener, *et al.*, they demonstrate that HMGB1 is necessary for neutrophil migration in sterile inflammation.^60^ Necrotic hepatocytes release HMGB1 which drives chemotaxis of neutrophils through the receptor for advanced glycation end-products (RAGE), activating sterile inflammation and amplifying liver injury^60^ (Fig. 4).

Structurally homogeneous HS oligosaccharides were recently evaluated for its protection effect against APAP-induced acute liver injury.^61^ In this study, a HS octadecasaccharide (18-mer HP, or hepatoprotective 18-mer, Table 1) was synthesized using the chemoenzymatic approach (Fig. 4B). Mice treated with 18-mer-HP after APAP overdose had significantly healthier livers than APAP control mice as indicated by a lower plasma level of alanine aminotransferase. A series of target identification experiments were conducted to demonstrate that the 18-mer-HP targets to the HMGB1/RAGE axis. One line of evidence compares the protection efficacy with HMGB1 neutralizing antibody (α-HMGB1). It has been reported that α-HMGB1 protects against liver injury from APAP overdose through neutralizing the pro-inflammatory effect of HMGB1.^62^ We discovered that treatment of α-HMGB1 and 18-mer-HP offered very similar protection against APAP-induced liver injury. A combination treatment of α-HMGB1 and 18-mer-HP did not decrease ALT compared to either treatment alone, suggesting that both 18-mer-HP and α-HMGB1 achieve their hepatoprotection by targeting to the same biological process. Two additional lines of evidence to confirm that 18-mer-HP targets to HMGB1/RAGE axis are offered by the authors:^61^ 18-mer-HP diminishes the HMGB1-mediated neutrophil infiltration in mouse models; and 18-mer-HP's protection effect is lost in RAGE knockout mice. Interestingly, authors discovered that an anticoagulant 18-mer (18-mer AXa, Table 1) does not display hepatoprotection after APAP overdose. The loss of the protection effect of 18-mer AXa is attributed to the fact that the oligosaccharide impairs liver repairment. Fibrin is required to trigger liver regeneration process after APAP damage while the anticoagulant activity from 18-mer AXa eliminates the production of fibrin.

**Table 1:** Sequences of HS oligosaccharides described in this review

Name	Abbreviated saccharide sequence	Functions
18-mer-HP	GlcNS-GlcA-GlcNS-IdoA2S-GlcNS-IdoA2S-GlcNS-IdoA2S-GlcNS-IdoA2S-GlcNS-IdoA2S-GlcNS-IdoA2S-GlcNS-IdoA2S-GlcNS-GlcA-pNP	Protect against acetaminophen-induced liver damage
18-mer-AXa	GlcNS6S-GlcA-GlcNS3S6S-IdoA2S-GlcNS6S-IdoA2S-GlcNS6S-IdoA2S-GlcNS6S-IdoA2S-GlcNS6S-IdoA2S-GlcNS6S-IdoA2S-GlcNS6S-IdoA2S-GlcNS6S-GlcA-pNP	Has anticoagulant activity, but no hepatoprotection effect against acetaminophen-induced liver damage
12-mer-1	GlcNS6S-GlcA-GlcNS3S6S-IdoA2S-GlcNS6S-IdoA2S-GlcNS6S-IdoA2S-GlcNS6S-IdoA2S-GlcNS6S-GlcA-pNP	Protect against liver damage caused by ischemia/reperfusion damage
12-mer-3	GlcNS6S-GlcA-GlcNS6S-IdoA2S-GlcNS6S-IdoA2S-GlcNS6S-IdoA2S-GlcNS6S-IdoA2S-GlcNS6S-GlcA-pNP	Binds to HMGB1, but has no protection against ischemia/reperfusion damage
6-mer-AXa	GlcNS6S-GlcA-GlcNS3S6S-IdoA2S-GlcNS6S-GlcA-pNP	Has anticoagulant activity but does not bind to HMGB1

The 18-mer-HP treatment has a potential benefit for late-presenting APAP overdose patients by offering a wider therapeutic window than the standard of care, treatment with N-acetyl cysteine (NAC). NAC treatment is only effective if given within 8 hours after APAP ingestion in patients.^63^ The study found that delay of treatment with 18-mer-HP is still protective in mice. Delay treatment with 18-mer-HP at 3-hour post APAP fully protect mice, while NAC was unable to provide any protection compared to untreated mice. This significant increase in survival with 18-mer-HP treatment compared to the standard of care confirms that 18-mer-HP extends the therapeutic window after a lethal APAP overdose.

### Using anticoagulant HS oligosaccharides to protect liver damage against ischemia–reperfusion (I/R)

Thromboinflammation is the result of crosstalk between the innate immune system and the coagulation cascade after disruptions in the vascular system.^64^ Thrombosis and inflammation are traditionally viewed as separate processes that complement each other, but growing evidence supports the relationship between thrombosis and inflammation stimulating and reinforcing one other especially in sepsis, ischemia reperfusion injury, trauma, and severe burns.^65^ These cases support the role of thrombosis and thromboinflammation as mediators of sterile inflammation. The endothelial glycocalyx is an important regulator of thromboinflammation by acting as an anti-coagulant and anti-inflammatory barrier. Therefore, disruption of the glycocalyx is central to thromboinflammation.

In liver ischemia/reperfusion injury, nitric oxide decreases, ATP stores decrease, and the vasculature narrows.^66^ Hepatocyte mitochondria that are deprived of oxygen produce reactive oxygen species (ROS) which leads to cell death.^67^ Liver sinusoidal endothelial cells (SECs) become activated and upregulate adhesion molecules including von Willebrand factor (VWF), P-selectin, and intercellular adhesion molecule 1 (ICAM-1).^65^ Tissue factor (TF) lies beneath the endothelium and is exposed during vessel wall injury, where it can serve as a potent activator of coagulation by generating thrombin (FIIa).^65^ Thrombin is also generated by the contact pathway, which can be initiated by exposure of nucleic acids from damaged cells or inorganic phosphates from platelets. Thrombin's coagulation role entails activation of platelets to a procoagulant state and fibrin generation, while its inflammatory role entails activation of endothelial cells. Activated platelets promote neutrophil recruitment to the activated endothelial cells. Activated platelets can also secrete HMGB1 even though they lack a nucleus.^68^ Platelets also express HMGB1 receptors, RAGE, TLR2, TLR4, and TLR9, on their surface, suggesting that HMGB1 can stimulate both platelet and neutrophil activation, further promulgating thromboinflammation. HMGB1, either from necrotic hepatocytes and/or activated platelets, can activate Kupffer cells which releases TNF-α and chemokines, further propagating neutrophil recruitment.^65^ Neutrophils contribute to hepatocyte death by releasing ROS.

In a recent publication, synthetic HS oligosaccharides decreased liver ischemia/reperfusion injury in mice.^69^ The combination of synthetic HS's anticoagulant activity *via* FXa inhibition and anti-inflammatory activity *via* HMGB1 inhibition makes it a prime anti-thromboinflammation therapeutic. This hypothesis was proved using several synthetic HS oligosaccharides with different biological functions: 12-mer-1 (Table 1) has both anticoagulant and HMGB1 binding ability; 12-mer-3 (Table 1) has only HMGB1 binding ability; 6-mer-AXa has only anticoagulant activity. Only 12-mer-1 treatment in the I/R model decreased plasma alanine transamidase (ALT), hepatic necrosis, and neutrophil infiltration. Interestingly, a combination treatment of 12-mer-3 and 6-mer-AXa (Table 1) also decreased liver injury compared to either compound alone. These results demonstrated that I/R injury is decreased in the presence of an anticoagulant and an anti-inflammatory HS(s). The findings further demonstrate the use structurally homogeneous oligosaccharides to probe the functions of HS *in vivo*.

## Conclusions

HS is an essential glycan for animals and human physiology. Structural complexity of HS isolated from natural sources complicates the investigation of the roles of HS in biological processes. The chemoenzymatic method for the synthesis of structurally defined HS oligosaccharides has made the oligosaccharides available to the research community, removing a key barrier for advancing HS research. The chemoenzymatic synthesis enables preparing diverse structures, amenable for generating HS oligosaccharide libraries. The method is also fully capable of carrying out scale-up synthesis for the development of HS-based anticoagulant. The utilities of the homogeneous oligosaccharides synthesized by the chemoenzymatic method are emerging from our work. In one example, the method offers a potential to prepare synthetic heparin cost-effectively to improve the safety of heparin drug. In another example, the oligosaccharides are used to discover the anti-inflammatory activity of HS in diseases models. In our view, broad access of the oligosaccharides to biologists will accelerate the discovery of biological functions of HS.

## Conflicts of interest

JL is a founder and chief scientific officer for Glycan Therapeutics. YX is also a founder for Glycan Therapeutics. Other authors declare no competing interest. Dr Jian Liu's lab at the University of North Carolina has received a gift from Glycan Therapeutics to support the research in glycoscience.
